# Analgesic effect of methadone constant rate infusion is not superior to fentanyl constant rate infusion in dogs after spinal surgery: a prospective, randomized, blinded, clinical trial

**DOI:** 10.3389/fvets.2026.1873178

**Published:** 2026-09-23

**Authors:** Alexandra Friederike Schütter, Sabine Beate Rita Kästner, Ben Prause, Julia Tünsmeier

**Affiliations:** Small Animal Hospital, University of Veterinary Medicine Hannover, Hannover, Germany

**Keywords:** Glasgow Composite Measure Pain Scale, opioid, pain, spinal disease, von Frey filaments

## Abstract

**Objectives:**

This study aimed to compare the analgesic efficacy of methadone and fentanyl infusions at a 50:1 dose rate in dogs after spinal surgery.

**Methods:**

In a prospective, randomized, blinded clinical trial, 42 dogs with intervertebral disk disease were evaluated at 1, 2, 4, 18, 24, 42, 48, 66, 72, 90, and 96 h after extubation. Based on the treatment groups, the dogs received a presurgical bolus of 0.25 mg/kg methadone or 5 μg/kg fentanyl, followed by an intraoperative and postoperative opioid infusion. In the methadone (M) treatment group, the dogs received 0.2 mg/kg/h methadone, which was reduced stepwise to 0.05 mg/kg/h. In the fentanyl (F) treatment group, the initial dose of 5 μg/kg/h was reduced stepwise to 1 μg/kg/h. Assessments at each time point included a simple sedation score, vital parameters, pain scoring using the Glasgow Composite Measure Pain Scale (CMPS-SF), and sensory testing with von Frey filaments. Multiple Mann–Whitney tests were used for the analysis of CMPS-SF scores and sedation scores. Vital parameters, between and within the groups, were analyzed using mixed-effects analysis and Dunnett’s multiple comparison test. The need for rescue analgesia was assessed via survival analysis. The level of significance was set to α = 5%.

**Results:**

In the postoperative period, six dogs in group M and four dogs in group F required rescue analgesia. CMPS-SF scores were not different between the groups M and F and did not change significantly over time within the treatment groups. Tactile sensory thresholds were not different between the groups M and F.

**Clinical significance:**

Under the dosing regimens investigated, methadone did not provide superior postoperative analgesia compared to fentanyl. From a clinical perspective, both drugs provided effective postoperative analgesia in this heterogeneous cohort of dogs following spinal surgery.

## Introduction

Intervertebral disk disease is the most common diagnosis in dogs presented as non-ambulatory emergencies (1). Affected dogs suffer from pain (2), which might include nociceptive, inflammatory, and neuropathic components (3). Several analgesic protocols have been documented to address the different layers of complex pain that veterinary patients might suffer after spinal surgery. Currently, multimodal analgesic protocols, including opioids, local anesthetics, and ketamine, are used to treat this kind of pain (4–10).

Methadone, a µ-opioid receptor agonist and an N-methyl-D-aspartate (NMDA) receptor antagonist, contributes to adequate postoperative analgesia in dogs with intervertebral disk disease (8–12). Furthermore, the antinociceptive properties of methadone are enhanced via reuptake inhibition of serotonin (13) and most likely norepinephrine (13). Moreover, methadone can cause a non-competitive blockade of α3β4 nicotinic receptors (14). Due to its multiple mechanisms of action, methadone may provide multimodal analgesia as a single agent in dogs with mixed pain syndromes, such as those associated with intervertebral disk disease.

In human patients experiencing severe neuropathic pain associated with head and neck cancer, methadone treatment was associated with significantly lower numeric rating scale scores compared to fentanyl (15). Furthermore, the rate of clinical success—defined as a greater than 50% improvement—was higher in the methadone group (15).

Despite the theoretical benefits of methadone, the clinical effect of these extra modes of action might not be clinically significant. Subcutaneous methadone and transdermal fentanyl showed the same analgesic effectiveness in dogs after spinal surgery when used in combination with pregabalin (10).

However, the addition of pregabalin could have masked group differences. Furthermore, with repeated bolus administration, plasma concentrations vary significantly, which may influence efficacy and increase dose-dependent side effects, respectively.

Stable plasma concentrations of methadone and acute antinociceptive effects have been reported following a constant rate infusion (CRI) of 0.1 mg/kg/h in healthy laboratory Beagle dogs (16). This study showed that a steady state was reached after 12 h of methadone CRI and that no accumulation occurred during a 72-h observation period (16). The occurrence of opioid-induced hyperalgesia (OIH) during or after treatment in humans is documented. Fentanyl is discussed as being one potential trigger of this phenomenon, whereas methadone can be used to treat OIH (17, 18). Evidence for canines currently is lacking, but the prevention of OIH might be an additional benefit in using methadone instead of fentanyl for postoperative analgesic treatment.

Therefore, this study aimed to compare the analgesic efficacy of a methadone CRI to a fentanyl CRI in dogs after spinal surgery. The hypothesis was that a methadone CRI starting before surgery would provide superior pain relief to a fentanyl CRI starting before surgery, as determined by clinical pain scoring, rescue analgesia requirements, and skin sensitivity testing.

## Materials and methods

### Ethical review and study design

The study design was reviewed in accordance with the German Animal Welfare Act and approved by the Lower Saxony State Office for Consumer Protection and Food Safety (LAVES) (reference number 18A 318).

This study was designed as a randomized, prospective, clinical trial. Using commercially available software (GraphPad QuickCalcs), the dogs were randomly assigned to one of the two treatment groups. A veterinarian not involved in patient handling and evaluation prepared the study drugs, which were labeled “study opioid.” The anesthetist in charge, during imaging, surgery, and postoperative pain evaluations, together with imagers, surgeons, and ward nurses, was not aware of the allocated study groups. The neurologist in charge was not blinded to treatment but was not involved in patient assessment for this study. Patients were recruited from 10 July 2018 to 4 November 2019.

### Treatments

The methadone (M) group received preoperatively 0.25 mg/kg methadone (Comfortan 10 mg/mL, Eurovet Animal Health B. V., The Netherlands) intravenously (IV), followed by an intraoperative constant rate infusion of 0.2 mg/kg/h methadone. This was continued postoperatively with a rate of 0.1 mg/kg/h methadone for 72 h and then reduced to 0.05 mg/kg/h methadone for the last 24 h of the trial. The fentanyl (F) group received a preoperative bolus of 5 μg/kg fentanyl (Fentanyl Janssen 0,05 mg/mL, Janssen-Cilag GmbH, Germany) IV. Infusion rates in this treatment group were 5 μg/kg/h fentanyl intraoperatively and 2 μg/kg/h fentanyl postoperatively for 72 h, followed by 1 μg/kg/h for the last 24 study hours.

The dogs were privately owned, regular patients of the Small Animal Clinic, University of Veterinary Medicine Hannover, Foundation. Written informed owner consent was obtained before study enrollment. The inclusion criteria were as follows: dogs that were aged > 12 months and had thoracolumbar or cervical disk disease requiring surgical intervention. The exclusion criteria were as follows: dogs that were aged < 12 months, dogs in which pain scoring was not possible (e.g., highly aggressive demeanor), dogs with paraplegia without deep pain sensation (Sharp and Wheeler grad 5), dogs requiring revision surgery during the course of the study, dogs intolerant to any of the drugs used in the trial, dogs with any chronic disease under analgesic therapy, pregnant or lactating bitches, and dogs in which placement of an intravenous catheter was not possible without deep sedation.

### Anesthesia

In addition to one opioid (methadone or fentanyl according to the treatment group), the dogs received 10 μg/kg glycopyrronium (Robinul®, 0.2 mg/mL, Riemser Pharma GmbH, Germany) subcutaneously before anesthesia induction. Anesthesia was administered IV with 0.5 mg/kg diazepam (Ziapam®, 5 mg/mL, Ecuphar GmbH, Germany) and propofol (Narcofol, CP-Pharma, Burgdorf, Germany) to effect. Tracheas were intubated using a cuffed endotracheal tube. Anesthesia was maintained using isoflurane (Isoflurane CP, CP-Pharma, Burdorf, Germany) in oxygen, delivered to effect via a circle breathing system. Respiratory rate (RR), expiratory carbon dioxide, inspiratory and expiratory concentrations of anesthetic agent, electrocardiogram, oscillometric blood pressure, percentage of oxygenated hemoglobin, pulse rate, and body temperature were monitored using a multiparameter anesthesia monitor and documented every 5 min (data not shown).

Depending on the treatment group, intraoperative rescue analgesia of either 0.2 mg/kg methadone or 5 μg/kg fentanyl was administered IV if the heart rate (HR) increased >20% compared to the pre-incisional heart rate with a concomitant rise in blood pressure. To keep the blinding of the anesthetist patent, a second veterinarian not blinded to treatment would draw up and apply rescue analgesia if necessary. Multiple doses of rescue analgesia were allowed during the procedures.

### Patient assessment

After completion of the surgery, the dogs were allowed to recover in a warm, quiet kennel with reduced lights under the direct supervision of the researcher. Removal of the endotracheal tube was set as time point 0. Patient assessment was also performed at 1, 2, 4, 18, 24, 42, 48, 66, 72, 90, and 96 h after extubation. At each of these time points, measurements were taken in the same order. First a simple sedation score ranging from 0 to 3 was determined. In this score, 0 represented an awake dog, 1 represented a drowsy dog that reacted promptly, 2 represented a dog that was sedated and recumbent with delayed reaction to manipulation, and 3 represented a dog that was deeply sedated and only reacted to forceful manipulation. If the sedation score was ≥ 2, indicating that the dog was still sedated, no further measurements were taken, and the dog was reevaluated 1 h later. In case the sedation score was less than two, assessments were performed. These included respiratory rate by visual count, heart rate by chest auscultation, rectal temperature via a digital thermometer, and oscillometric blood pressure measurement (petMAP graphic II Ramsey Medical, Inc., USA) at one thoracic leg or the tail. Pain evaluation was performed using the short form of the Glasgow Composite Measure Pain Scale (CMPS-SF). According to Reid et al. (19), section B was excluded in non-ambulatory dogs.

A set of classical von Frey filaments was used in ascending order to test skin sensitivity (tactile sensory threshold) bilaterally at 3, 5, and 10 cm distances from the surgical suture line. Filaments were applied orthogonally to the skin surface until they either bent maximally or the dog reacted purposefully (movement of the head, elusion, or evasion). If the patient was not responsive to the stimulus, the next thicker filament was deployed. A minimum time of 10 s was allowed between testing different filaments in one location. This procedure was continued until a positive response could be elicited or the thickest filament was reached.

### Rescue analgesia and disqualifying criteria

The need for postoperative rescue analgesia was indicated by a CMPS-SF score of ≥ 5 in non-ambulatory and ≥ 6 in ambulatory dogs (19). Rescue analgesia triggered immediate termination of the study. Data of these dogs were excluded from further analysis for all time points after rescue analgesia. Independent of the treatment group, initial rescue analgesia consisted of 0.1 mg/kg methadone IV. If this was not sufficient, further methadone or other analgesics were administered until a CMPS-SF ≤ 5 or 6 was reached, respectively.

Further disqualifying criteria in the post-surgical period in this clinical trial were as follows: severe bradycardia (< 40 beats per min), severe respiratory depression (< 4 breaths per min), severe and persistent hypothermia (< 36.0 °C) or hyperthermia (> 40.0 °C), constipation, profound sedation after study drug application, and distinct and non-self-limiting dysphoria for longer than 15 min. All non-analgesic medications (e.g., dibenzyran, myocholine, omeprazole, sucralfate, and metoclopramide) were at the discretion of a European College of Veterinary Neurology resident under supervision. These veterinarians were not involved in this trial. In case they were unsure about study interference of drugs, they could always contact the study supervisor.

### Statistical analysis

*A priori* sample size calculation using a z-test (unpooled variance) with a power of 80%, an alpha of 5%, and an effect size of 0.8 (calculated for a difference in the CMPS-SF group means of 2 ± 2) indicated the need for 21 dogs in each treatment group. Statistical analysis was performed using MS Excel (Version 16.0), SAS (Version 9.4 M5), and GraphPad Prism (Version 10.5.0). Normal distribution of data was assessed using the Shapiro–Wilk test and visual evaluation of q–q plots. Demographic data were analyzed using descriptive statistical methods. Data of dogs reaching a disqualifying criterion were excluded from further analysis for all time points after they reached the disqualifying criterion.

Multiple Mann–Whitney tests were used for the analysis of CMPS-SF scores and sedation scores. Analyses of heart rates, respiratory rates, and blood pressures between the groups and within the groups compared to time point 0 (extubation) were performed using mixed-effects analysis and Dunnett’s multiple comparisons test. The need for rescue analgesia was evaluated using a survival analysis. The level of significance was set to α = 5%.

## Results

Demographic data were not different between the treatment groups and are demonstrated in Tables 1, 2.

**Table 1:** Demographic data of 42 dogs that received either methadone CRI or fentanyl CRI during and after spinal surgery.

	Methadone (*n* = 21)	Fentanyl (*n* = 21)
Female	4	3
Female neutered	5	4
Male	6	6
Male neutered	6	8
Age (month)	78 ± 32	89 ± 40
Weight (kg)	15.6 ± 9.8	11.5 ± 6.0

Age and weight are displayed as mean ± standard deviation.

**Table 2:** Distribution of breeds of dogs included in the methadone and fentanyl treatment groups.

	Methadone (*n* = 21)	Fentanyl (*n* = 21)
Crossbreed	5	7
Dachshund	5	4
French Bulldog	4	4
Beagle	3	1
Jack Russell Terrier	1	1
Maltese	2	0
Pinscher	0	1
Miniature Pinscher	0	1
Havanese	0	1
Cocker Spaniel	0	1
American Bulldog	1	0

Seven dogs in group M and three dogs in group F suffered from cervical disk disease. Fourteen dogs in group M and 18 dogs in group F had thoracolumbar disk disease.

The need for intraoperative rescue analgesia was not different between the treatment groups. In group M, six dogs with thoracolumbar disk disease and one dog with cervical disk disease needed additional methadone. In group F, 12 dogs with thoracolumbar disk disease and one dog with cervical disk disease needed additional fentanyl. The median (range) number of intraoperative rescue boluses per dog was 0 (0–4) in group M and 1 (0–3) in group F.

In both treatment groups, sedation scores were less than two at all time points.

CMPS-SF scores were analyzed for cervical and thoracolumbar disk disease separately. As no group differences could be found, dogs with cervical and thoracolumbar disk disease were pooled for further analysis. At no time point, CMPS-SF scores were different between the treatment groups M and F (Figure 1). Within each treatment group, no significant differences between time point 0 (extubation) and following time points were detectable. All dogs required assistance for locomotion during the postoperative period studied; therefore, section B of the CMPS-SF was omitted.

**Figure 1:**
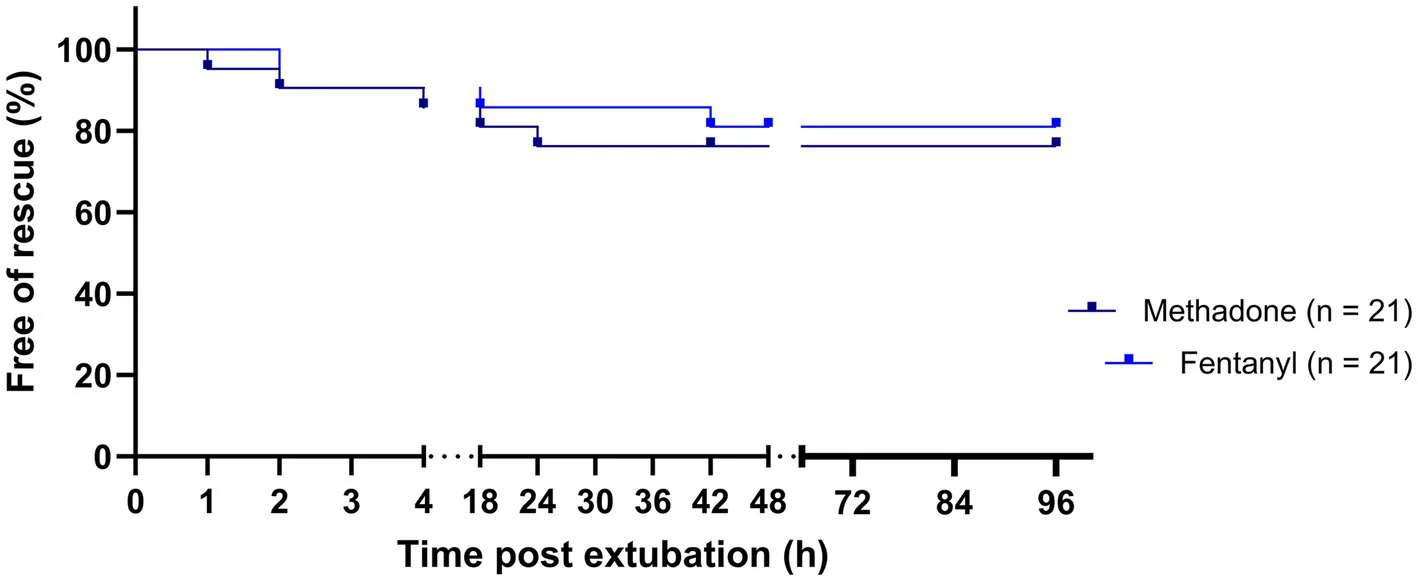
CMPS-SF pain scores over time for dogs in the methadone group (dark blue) (starting at 0.1 mg/kg/h) and in the fentanyl group (light blue) (starting at 2 μg/kg/h). After rescue analgesia, the dogs were excluded from further analysis. Boxes contain 50% of the data, the horizontal line within the box indicates the median, and the whiskers indicate the minimum and maximum values.

Postoperatively, six dogs in group M (two with cervical disease, four with thoracolumbar disease) and four dogs in group F (all with thoracolumbar disease) reached the CMPS-SF rescue cutoff of ≥5 and required rescue analgesia. The need for postoperative rescue analgesia was statistically not different between the treatment groups (*p* = 0.67) (Figure 2).

**Figure 2:**
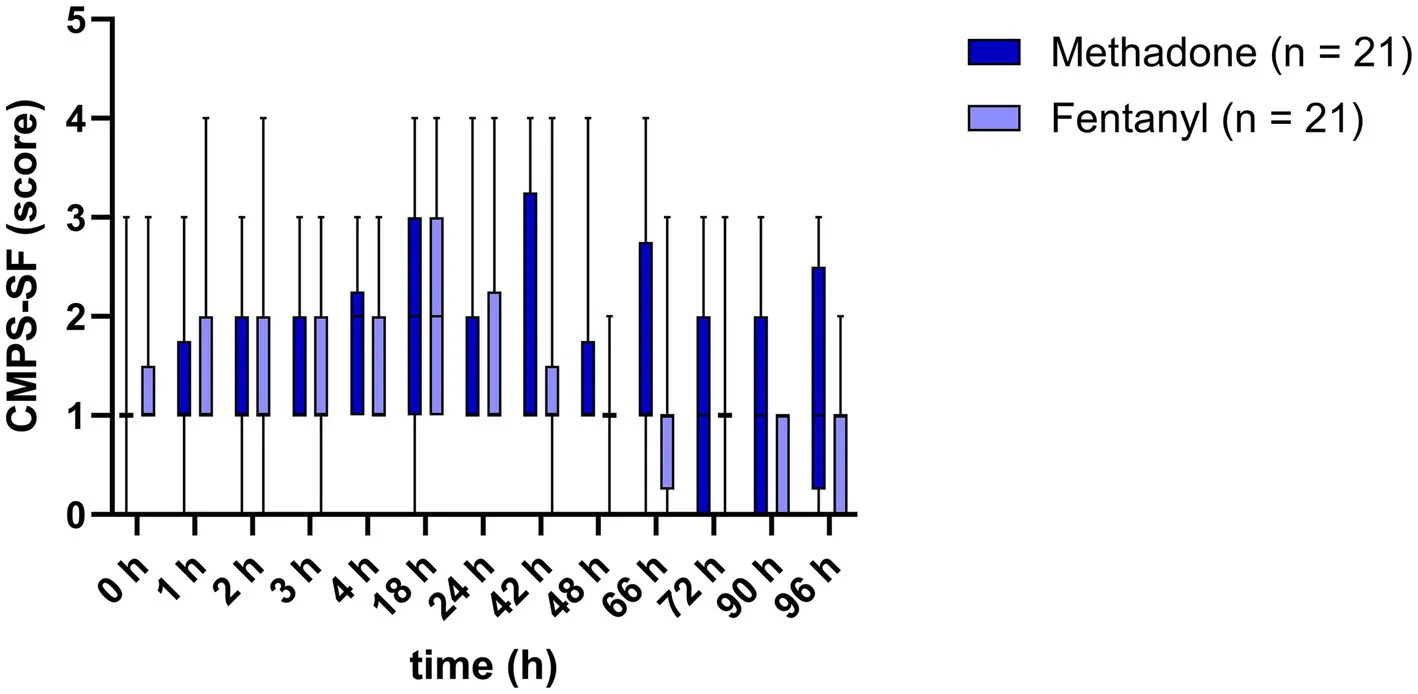
The Kaplan–Meier curve indicates the timing of rescue analgesia in 21 dogs in the methadone group (dark blue) (starting at 0.1 mg/kg/h) and 21 dogs in the fentanyl group (light blue) (starting at 2 μg/kg/h) over a 96-h postoperative observation period.

At no time point, tactile sensory thresholds were different between the treatment groups M and F (Figure 3).

**Figure 3:**
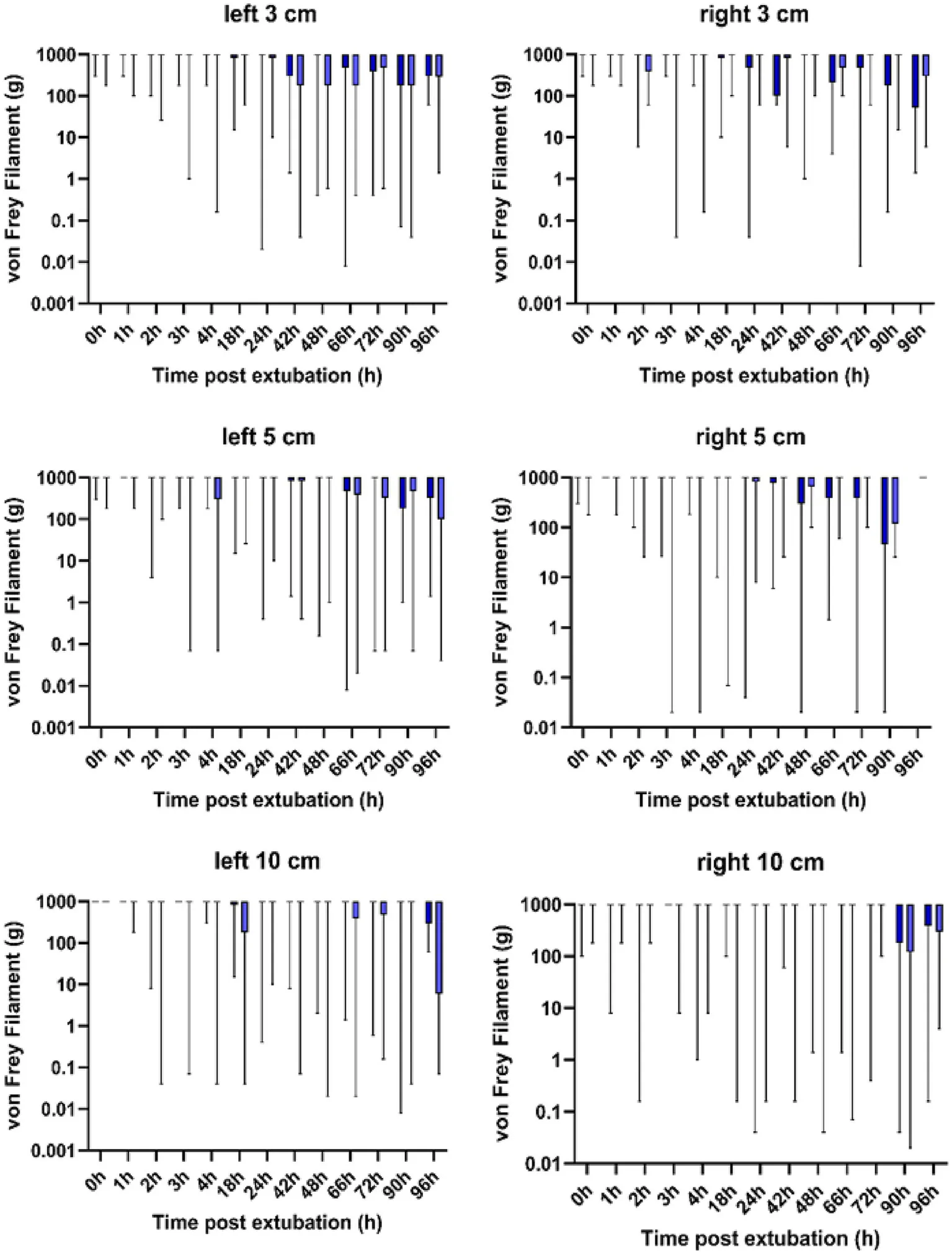
Tactile sensory thresholds were evaluated using von Frey filaments over time for 21 dogs in the methadone group (dark blue) (starting at 0.1 mg/kg/h) and 21 dogs in the fentanyl group (light blue) (starting at 2 μg/kg/h) over a 96-h postoperative observation period. After rescue analgesia, dogs were excluded from further analysis. The reactions of the left and right sides of the body at 3, 5, and 10 cm distances to the surgical wound are shown. Boxes contain 50% of the data, the horizontal line within the box indicates the median, and the whiskers indicate the minimum and maximum values.

The overall reaction to von Frey stimulation showed large individual variation. In 79.2% of measurements, no reaction was detectable at all. In an additional 12.9% of tests, only filaments larger than 100 g could cause a reaction to stimulation. At time point 42 h, the disqualifying criterion hypothermia <36.0 °C was reached in one dog in group M. No further disqualifying criteria were reached in any treatment. Furthermore, no major adverse effects were noticed.

Observed minor adverse effects are listed in Table 3.

**Table 3:** Adverse effects occurring during a 96-h postoperative observation period in the treatment groups methadone (starting at 0.1 mg/kg/h) and fentanyl (starting at 2 μg/kg/h).

	Methadone	Fentanyl
Hypothermia (< 37.0°C)	5	0
Bradycardia (< 50/min)	4	1
Dysphoria (time 0–4 h)	3	3
Dysphoria (time 4–96 h)	2	1
Vomitus	0	1
Mydriasis	1	0
Hyperthermia (> 39.3 °C)	0	1

During the whole study period, HR was lower in group M than in group F, but significance was only reached at time points 3 and 4 h (Table 4). Blood pressure, respiratory rate, and body temperature were not different between the treatment groups (Table 4).

**Table 4:** Vital parameters in the treatment groups M (methadone) (starting at 0.1 mg/kg/h) and F (fentanyl) (starting at 2 μg/kg/h) during a 96-h observation period displayed are mean and standard deviation for each parameter.

		0 h	1 h	2 h	3 h	4 h	18 h	24 h	42 h	48 h	66 h	72 h	90 h	96 h
HR /min	M	83 ± 20	85 ± 29	86 ± 25	77 ± 21*	80 ± 20*	79 ± 23	80 ± 31	73 ± 23	77 ± 18	84 ± 20	76 ± 21	89 ± 28	79 ± 18
	F	105 ± 40	99 ± 27	105 ± 23	111 ± 31*	109 ± 28*	98 ± 36	97 ± 23	92 ± 24	90 ± 21	92 ± 22	94 ± 23	93 ± 22	86 ± 19
RR /min	M	22 ± 11	29 ± 15	27 ± 13	27 ± 13	31 ± 19	28 ± 11	27 ± 7	29 ± 14	26 ± 16	23 ± 6	25 ± 12	34 ± 19	24 ± 6
	F	25 ± 9	32 ± 14	31 ± 11	38 ± 14	37 ± 13	25 ± 12	37 ± 23	31 ± 13	33 ± 14	29 ± 11	30 ± 12	25 ± 12	23 ± 6
SAP mmHg	M	146 ± 20	148 ± 25	154 ± 21	153 ± 25	152 ± 24	164 ± 16	163 ± 24	162 ± 31	159 ± 25	169 ± 24	169 ± 20^#^	168 ± 16	163 ± 24
	F	152 ± 19	164 ± 23	159 ± 26	168 ± 21	162 ± 26	161 ± 23	173 ± 23^#^	173 ± 24^#^	171 ± 20^#^	165 ± 24	160 ± 22	165 ± 22	170 ± 19
MAP mmHg	M	100 ± 20	104 ± 23	108 ± 20	106 ± 20	109 ± 23	117 ± 25^#^	116 ± 25	114 ± 26^#^	115 ± 23	124 ± 19^#^	118 ± 13^#^	118 ± 14^#^	113 ± 17^#^
	F	106 ± 15	113 ± 23	111 ± 22	113 ± 19	113 ± 25	113 ± 21	119 ± 22^#^	119 ± 24	118 ± 16^#^	111 ± 20	108 ± 21	111 ± 17	119 ± 13
DAP mmHg	M	73 ± 20	74 ± 20	80 ± 20	79 ± 19	79 ± 18	89 ± 22^#^	89 ± 27	85 ± 22	88 ± 24	92 ± 21^#^	87 ± 14	89 ± 13^#^	83 ± 17
	F	80 ± 16	83 ± 22	84 ± 22	85 ± 24	85 ± 25	85 ± 23	88 ± 24	88 ± 24	89 ± 19	80 ± 15	79 ± 19	81 ± 14	89 ± 13
Temp. °C	M	36.4 ± 1.4	36.8 ± 0.9	37.4 ± 0.8	37.5 ± 0.8	37.7 ± 0.7	37.6 ± 0.9	37.8 ± 0,7	37.6 ± 0.9	37.6 ± 0.7	38.0 ± 0.7	38.0 ± 0.6	38.2 ± 0.4	38.2 ± 0.2
	F	36.4 ± 1.4	37.3 ± 0.9	37.8 ± 0.8	38.0 ± 0.8	38.1 ± 0.7	38.3 ± 0.7	38.3 ± 0.5	38.3 ± 0.4	38.3 ± 0.5	38.3 ± 0.5	38.5 ± 0.3	38.4 ± 0.5	38.2 ± 0.5

For calculation of the RR, panting was excluded. Significant differences between the treatment groups at a time point are indicated with *, and significant differences within a treatment group compared to time point 0 h (extubation) are indicated with ^#^. HR, Heart rate; RR, Respiratory rate; SAP, Systolic arterial pressure; MAP, Mean arterial pressure; DAP, Diastolic arterial pressure; Temp, Rectal body temperature.

## Discussion

Based on clinical pain evaluation using the CMPS-SF and skin sensitivity testing, both methadone and fentanyl CRIs provided sufficient analgesia for the majority of dogs throughout the 96-h postoperative observation period during this clinical trial. Therefore, the study hypothesis—that methadone mono-analgesia would provide superior postoperative pain relief compared to fentanyl mono-analgesia in dogs undergoing spinal cord surgery—could not be confirmed. This should not be interpreted as evidence of equivalent analgesic efficacy between the two treatments, as the study was designed and powered to detect superiority and not equivalence. With the current study design, it remains unclear whether the present findings reflect a true equivalence in analgesic efficacy or whether subtle differences were undetectable due to limitations of the measurement techniques or small, non-obvious disparities between the groups. The CMPS-SF was the primary tool used for patient evaluation, relying on canine behavioral assessment to estimate pain levels (19, 20). Even though the CMPS-SF has a clear structure, a behavioral-based pain assessment tool will never be fully objective and leaves some space for personal interpretation (21). This subjective component was minimized in the present trial because all assessments were performed by the same observer. Still, there is a possibility that this single observer did not realize nuances of canine behavior, which could have masked group differences. Due to the clinical nature of the trial and to minimize stress and discomfort for patients, multiple observers were not included. Assessment of skin sensitivity using von Frey filaments was included to evaluate a possible neuropathic component of pain. This might, for example, present as hyperalgesia and allodynia as well as hypoalgesia or hypoesthesia. For the d-methadone enantiomer, an anti-hyperalgesic effect has been described in laboratory rodents (22), which could be beneficial in dogs suffering from neuropathic pain. However, in both treatment groups, the majority of dogs responded to the thickest filaments only or not at all. A definitive reason for the lack of reaction cannot be ruled out by behavioral observations only. Either adequate analgesia or impairment of the innervation of the surgical area could be speculated. However, this resembles the results of a study comparing subcutaneous methadone and transdermal fentanyl following spinal surgery, in which 40% of dogs showed no or a late reaction to von Frey filament testing (10). Surgical nerve injury and spinal impairment of sensory function have been proposed as explanations for the lack of reactivity observed during the first days after surgery, before sensory alterations develop (10). These considerations also apply to this study. Compared to Schütter et al.’s study (2025) (10), the reactivity toward von Frey filament measurements during application of methadone or fentanyl CRI was even lower, which might be caused by higher or more stable plasma levels of the drugs. Sedation scores over the whole study period were less than two; thus, sedation as a reason for absent or late reactions can be excluded.

In concordance with the above mentioned study (10), a statistically non-significant trend toward reactions at thinner filaments was observed on the last days of the observation period. This might suggest a return of skin sensitivity. As mentioned by Schütter et al. (2025) (10), von Frey filaments seem not to be the ideal tool for evaluation of patients in the first days after spinal surgery. Skin sensitivity seems to increase from day 4 on; hence, von Frey filaments might be more helpful at later time points for the detection of neuropathic pain.

Methadone and fentanyl are full μ-receptor as well as κ- and δ-receptor agonistic opioids (23, 24). They are both classified as effective analgesics in dogs. Pain following spinal surgery is multifactorial and may include nociceptive, inflammatory, and neuropathic components (3). For methadone and fentanyl, reliable antinociceptive action (16, 25, 26) or reduction of anesthetic requirements (27, 28) is documented. In an experimental setting using healthy research Beagle dogs, stable plasma concentrations of methadone could be achieved after 12 h of a methadone CRI at 0.1 mg/kg/h (16). These plasma concentrations were associated with sustained thermal and mechanical antinociceptive effects (16). However, plasma concentrations do not necessarily reflect effect-site concentrations, which determine pharmacodynamic response. Consequently, stable plasma concentrations alone cannot be interpreted as proof of constant analgesic effect-site concentrations.

Currently, no effect-site equilibration constant (ke₀) has been established for methadone in dogs. Therefore, the extent of plasma–effect-site hysteresis cannot be quantified in this species. However, the concurrent demonstration of antinociceptive effects by Amon et al. (16) provides functional evidence that the achieved plasma concentrations were associated with persistent opioid activity.

Furthermore, dogs in this study received an intravenous loading dose before the initiation of the CRI. This minimized the initial distribution phase and facilitated more rapid attainment of therapeutically relevant tissue and effect-site concentrations.

During the early postoperative period, nociceptive pain likely might represent the predominant pain component. Likely this component was already adequately addressed by the opioid treatments applied. Therefore, the additional NMDA receptor antagonism and monoamine reuptake inhibition attributed to methadone may not have resulted in measurable clinical differences between the treatments.

At present, a direct study establishing equipotent doses of methadone and fentanyl in dogs is lacking. However, for both drugs, comparisons to morphine are available. These state that racemic methadone in dogs is 1.75 times more potent than morphine (29), and fentanyl is 75 to 125 times more potent than morphine (30).

Based on these comparisons of both opioids to morphine, a ratio of 50:1 was assumed to be equipotent and chosen for this study. This assumption, without a distinct study supporting it, is a limitation of this study. In case the doses were not equipotent, the lack of detectable treatment differences and adverse effects could be a dose-related effect. Nevertheless, used opioid doses are within a clinically accepted dose range for dogs. Therefore, the comparison of the chosen doses is still clinically significant.

Considering vital parameters and occurring side effects, only minor, non-statistically significant differences between the treatment groups could be noted. This likely will also be of no clinical relevance. Heart rates within both groups did not vary much over the whole study period but were statistically non-significantly lower in group M than in group F. For methadone and fentanyl, dose-dependent bradycardia (16, 27, 31–34) due to activation of vagal efferent neurons (35) is described. The magnitude of this possible bradycardia seems comparable between the two drugs, as for both drugs, heart rate reductions in dogs of up to an approximately 50% are described (27, 32, 33). In human medicine, the occurrence of tachyphylaxis or tolerance after fentanyl is documented (36, 37). In dogs receiving a methadone constant rate infusion over 3 days, no signs of tachyphylaxis were evident (16). So possibly, the development of tachyphylaxis in group F could be one explanation for slightly higher heart rates in this group.

Furthermore, clinically, the majority of dogs appear to have lower heart rates. Dogs in group M showed a trend toward higher body weights than dogs in group F. However, scientific evidence for lower heart rates in heavier dogs is currently lacking (38, 39). Unfortunately, these studies investigating the connection between heart rate and body mass did only evaluate body weight and not body conditioning score or a similar measure. However, breed-dependent or individual differences in vagal tone could also be responsible for the variability in heart rates.

This study was not designed to investigate the safety of opioid analgesia treatments used. The sample size was not calculated to assess safety aspects of the treatments. Therefore, only emerging side effects in this limited population of dogs can be described and should be interpreted with caution. One of the main side effects noted was mild to moderate self-limiting dysphoria in the early postoperative period (0–4 h, after extubation). The incidence of this unwanted effect was equally distributed in both treatment groups. High opioid plasma levels, resulting from the higher intraoperative infusion rates, or the influence of other drugs applied during premedication or surgery (e.g., benzodiazepines), can be considered as possible reasons for the dysphoria. Single dogs showed signs of slight dysphoria at a later time point during the rest of the study. This might be caused by drug accumulation due to slow or reduced opioid metabolization in these individual dogs.

Despite the small sample size and the risk of overinterpretation of the current findings, methadone seems to promote hypothermia more easily than fentanyl, as five dogs in group M had a decrease in body temperature below 37 °C.

Reduction in dogs’ body temperature following methadone application is well documented (16, 34, 40, 41). Interestingly, in one dog in group F, an increase in body temperature was detectable. Both effects could be mediated through either κ- or μ-opioid receptors (41). Although the main investigator was blinded to treatment allocation, the investigator’s clinical experience could have led to assumptions about group allocation when observing adverse effects in individual dogs. This might potentially have introduced an unconscious bias during patient evaluation.

The clinical nature of the current study causes some circumstances that can be considered as limitations. To picture the clinical routine, all eligible dogs were included in the trial independent of the duration or location of the spinal disease. As this study included referred clinical patients, analgesic treatment before referral was not standardized and was not consistently documented. Consequently, a potential influence of pre-referral analgesia on the early postoperative pain assessments cannot be entirely excluded. Furthermore, the sample size is too small to make any subgroup-specific comparisons. It cannot be predicted whether subgroup characteristics would influence the study outcome in a larger or more homogeneous sample of dogs (e.g., dogs with only thoracolumbar disk disease). Therefore, the observations only apply to the heterogeneous group of dogs studied here.

It is likely that preoperative pain levels or pain types of individual dogs differed due to different locations or chronicity of disease. Dogs were included regardless of the prior analgesic treatment, which included a non-steroidal anti-inflammatory drug or a corticosteroid. Although any analgesic or anti-hyperalgesic treatment was stopped after admission to the hospital, this could have influenced results, particularly during the interoperative or early postoperative period. The large heterogeneity of the studied canine cohort might reduce the ability to detect treatment-specific effects. Nevertheless, aiming to investigate efficacy and possible adverse effects of opioid infusions, this heterogeneous group of patients represents a realistic picture of the dog population suffering from spinal disease. To avoid other drugs masking the effects of methadone or fentanyl, a mono-analgesic protocol was used during the study. This protocol differed from routine clinical practice and included predefined disqualifying criteria and a rescue analgesia strategy. Based on the results of this study, both methadone and fentanyl administered as CRIs at the doses used appeared to be effective and well tolerated in the majority of dogs studied, regardless of prior treatment, disease location, or chronicity. Nevertheless, clinically including the opioid CRI in a multimodal analgesia plan rather than using them as mono-analgesia seems to be more reasonable. Intraoperative rescue analgesia was applied based on changes in heart rate and blood pressure. Even though this is widespread clinical practice, it is important to keep in mind that these parameters can be changed not only by nociceptive events. Therefore, they are not reliable indicators for the analgesic/antinociceptive properties of a treatment protocol. Postoperative need for rescue analgesia did lead to direct termination of the study for this dog. This practice could have influenced the remaining data of this study and introduced a selection bias. The results for analgesic efficiency based on only CMPS-SF levels might appear more positive than they really are. However, it was decided to use this approach as it provides information on if and in how many dogs opioid mono-analgesia is sufficient for pain control but allows rescue analgesia with other drugs in animals that were still painful under the opioid CRI. Furthermore, excluding dogs that required rescue analgesia could also have led to an underestimation of occurring adverse effects. Drug doses were chosen based on a study measuring plasma concentrations, which likely will differ from effect-site concentrations. However, chosen doses did produce antinociception in experimental trials, which can be interpreted as marker for an adequate effect-site concentration.

If no disqualifying criterion was reached, opioid CRIs during the course of the trial were altered according to a preset experimental scheme and not based on the results of pain scoring. This alteration from clinical practice was chosen to standardize drug application and to allow for easier group comparisons. However, in some dogs, this might have led to the application of higher opioid doses than needed, which could have caused some of the adverse effects documented. On the other hand, in both treatment groups, some dogs required additional analgesia. Therefore, for clinical practice, adapting opioid application based on pain scoring seems to be more sensible. Furthermore, true multimodal analgesia using more than one drug was not intended to be investigated in this study. However, due to the occurrence of mild to moderate adverse effects (dysphoria, bradycardia, and hypothermia) in some dogs, it appears to be more appropriate to use methadone or fentanyl infusions as basal analgesia in a multimodal analgesic plan instead of using them as mono-analgesia.

The sample size for this study was calculated to detect a clinically significant difference between the treatment protocols and was based on a rather large difference in CMPS-SF scores. Therefore, smaller differences might have remained unnoticed, as groups might have been too small to detect them.

A mono-analgesia using methadone constant rate infusion did not provide superior pain relief to fentanyl constant rate infusion. No advantage of a methadone infusion compared to a fentanyl infusion could be demonstrated in the studied population using the CMPS-SF and skin sensitivity testing.

Clinically, both opioid infusions provided effective postoperative analgesia in the majority of dogs included in this study following spinal surgery. No major adverse effects were observed. Therefore, both drugs seem to be a reasonable choice for analgesic treatment of a heterogeneous group of dogs after spinal surgery, but clinical application as part of a multimodal analgesia plan rather than as mono-analgesia appears to be more appropriate.

## Data Availability

The original contributions presented in the study are included in the article/Supplementary material, further inquiries can be directed to the corresponding author.
