# Supplementary material for: Analgesic effect of methadone constant rate infusion is not superior to fentanyl constant rate infusion in dogs after spinal surgery: a prospective, randomized, blinded, clinical trial
Source: Front Vet Sci. 2026 Sep 23;13:1873178. doi: 10.3389/fvets.2026.1873178 (PMC13643910; doi:10.3389/fvets.2026.1873178)
Supplement: Supplementary file 1 [file Data_Sheet_1.PDF]

Figure 1: CONSORT 2025 Flow Diagram

Flow diagram of the progress through the phases of a randomised trial of two groups (that is, enrolment, intervention allocation, follow-up, and data analysis)

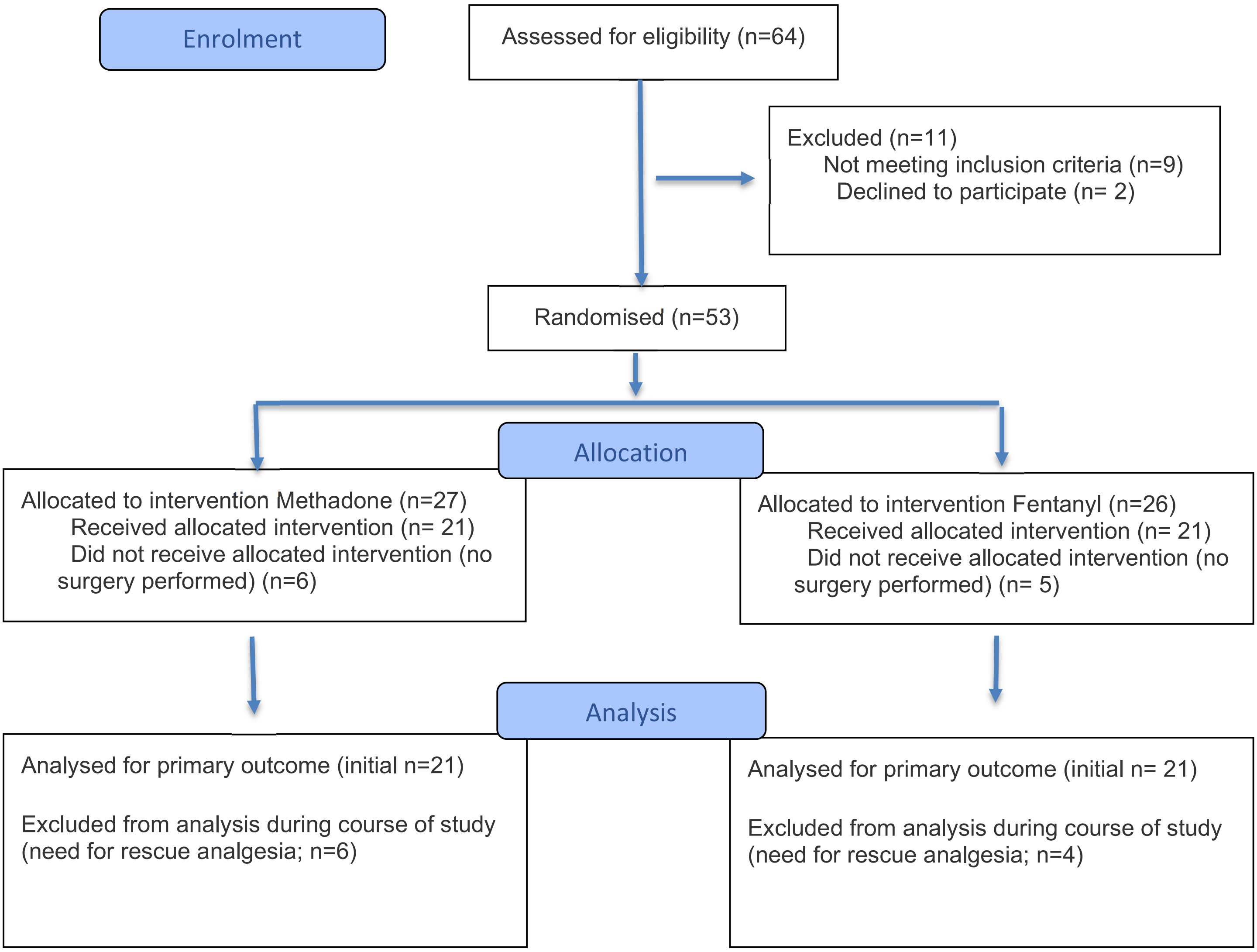

Citation: Hopewell S, Chan AW, Collins GS, Hróbjartsson A, Moher D, Schulz KF, et al. CONSORT 2025 Statement: updated guideline for reporting randomised trials. BMJ. 2025; 388:e081123. https://dx.doi.org/10.1136/bmj-2024-081123
© 2025 Hopewell et al. This is an Open Access article distributed under the terms of the Creative Commons Attribution License (https://creativecommons.org/licenses/by/4.0/), which permits unrestricted use, distribution, and reproduction in any medium, provided the original work is properly cited.
